# Too Much or Not Enough: The Role of *mprF* in Regulating Overall Phospholipid Content

**DOI:** 10.1128/mbio.03527-22

**Published:** 2023-04-06

**Authors:** Elizabeth M. Fozo

**Affiliations:** a Department of Microbiology, University of Tennessee, Knoxville, Tennesse, USA

**Keywords:** *Enterococcus faecalis*, lipidomics, lysyl-phosphatidylglycerol, glucosyl-lipids

## Abstract

Despite their fundamental role in defining cells, lipids and the contributions of specific lipid classes in bacterial physiology and pathogenesis have not been highlighted well. Enterococcus faecalis, a commensal bacterial and major hospital-acquired bacterium, synthesizes only a few known phospholipids. One of these variants, lysyl-phosphatidylglycerol, is critical for surviving cationic antimicrobial peptides, but its consequence on overall membrane composition and cellular properties has not been thoroughly examined. A recent study by Rashid et al. examines how loss of this lipid class results in an overall shift in total lipid composition and the consequential impacts on the global transcriptome, cellular growth, and secretion. They demonstrate the plasticity of the enterococcal lipidome to reprogram itself to allow for optimal function. With the significant improvements in multiple technological areas, this study, and others like it, provide a template for deciphering the critical function of lipids in all aspects of bacterial physiology.

## COMMENTARY

One of the first lessons in an introductory biology class is that a cell is surrounded by a phospholipid membrane, designed to separate internal processes and components from the external environment. However, the synthesis of this essential, foundational component of all cells is not discussed except in some more advanced courses. There is rarely a discussion as to how the membrane composition, especially the lipids, will dramatically shape cellular processes such as energy production, cellular signal transduction, and beyond. This is also reflective, to some extent, of how attention to the field has ebbed and flowed over the decades. The foundational work unraveling phospholipid synthesis began in earnest in the 1950s and continued for several decades, detailing specific biochemical processes and mapping the genes encoding such enzymes. Much of this pioneering and on-going work in bacteria was based on two model organisms: Escherichia coli, representing the Gram-negative species, and Bacillus subtilis, representing the Gram-positive species. Researchers also cataloged the membrane composition of a variety other bacterial species, including many human pathogens. Studies correlating membrane composition to its functionality in shaping cellular physiology, though, were not pursued in depth across most species. With the development of molecular and genetic tools beginning in the 1970s and 1980s, many researchers interested in bacterial pathogens focused their efforts on the identification of protein virulence factors, despite many unknowns remaining about the basic cellular biology of these organisms.

There has been a massive resurgence of interest in membrane lipid synthesis and how lipid composition shapes cellular processes in recent years. Much of this can be attributed to the increased sensitivity of mass spectrometry (the field of lipidomics) and advances in comparative genomics and metabolic mapping. Combining these technological improvements with traditional and careful microbial physiology to understand how lipid composition shapes bacterial cells, is exemplified in the article “Depleting cationic lipids involved in antimicrobial resistance drives adaptive lipid remodeling in Enterococcus faecalis,” by Rashid et al. (1). Within this article, the authors delve deep into the lipidome of E. faecalis, and specifically highlight the contributions of the phospholipid lysyl-phosphatidylglycerol to global features of its membrane and overall cellular physiology.

E. faecalis is a nosocomial pathogen, capable of inhabiting a variety of habitats. While it is an intestinal commensal of mammals, it is also a major contributor to hospital-acquired infections, and some isolates are critical for the fermentation of specific cheeses and sausages. Acclimation to so many different environments requires the organism to maintain the structure and functionality of its membrane to ensure proper energetic processes, transport of nutrients, efflux of waste, cell signaling, etc. Yet E. faecalis does not produce the variety of phospholipids found in many bacterial species. To date, it is known only to produce phosphatidylglycerol and two derivatives, lysyl-phosphatidylglycerol and cardiolipin (2–5). Glucosyl-lipids are also produced which can result in the formation of lipoteichoic acid. Despite this paucity, the organism “works with what it’s got” to successfully navigate changing environments.

Phosphatidylglycerol (PG), the predominant phospholipid of the membranes of enterococci and other Gram-positive pathogens, is anionic and contributes to the overall negative charge of the cell. The immune system exploits this via the production of cationic antimicrobial peptides that can damage or kill these pathogens. As was initially described in Staphylococcus aureus (6), E. faecalis utilizes MprF (multiple peptide resistance factor) which adds lysine to the lipid, forming lysyl-phosphatidylglycerol (L-PG), to reduce the overall membrane charge. Work by Bao et al. noted that in E. faecalis, there were two *mprF* genes, designated *mprF1* and *mprF2* (7). Their work, using two-dimensional thin-layer chromatography of deletion strains, concluded that deletion of *mprF2* resulted in the complete loss of L-PG, and work by my group and our collaborators confirmed these findings via a mass-spectrometry approach (5). Critically, Bao et al. and Kandaswamy et al. demonstrated the loss of L-PG resulted in increased sensitivity to cationic antimicrobial peptides (7, 8).

The work within by Rashid et al. does a thorough and careful analysis to conclude how loss of L-PG impacts the global lipidome and enterococcal physiology. Loss of L-PG would be expected to result in a build-up of the precursor, phosphatidylglycerol (PG). However, this does not occur: PG species are overall reduced in the Δ*mprF2* strain. My group also observed the same phenomena in a separate study when examining the lipidome of a Δ*mprF2* strain. Given that PG is also a precursor for cardiolipin (CL) which E. faecalis produces, an alternative possibility is that CL species would be increased in response to loss of L-PG production: in our work, total CL species were unchanged between the wild type and Δ*mprF2* strains (5).

What could be compensating for the dramatic decrease in phosphatidylglycerol? Both studies reveal that glucosyl-lipids are filling this void. Diglucosyl-diacylglycerol (DGDAG) lipids form the lipid anchor for LTA construction. DGDAGs are synthesized, in part, from PG. In particular, the glycerophosphate headgroup of PG is removed and then added to the growing LTA resulting in diacylglycerols (DAGs) within the membrane (9, 10). Rashid et al. observed a significant accumulation of DGDAG species in the Δ*mprF2* strain, and we noted far greater amounts of DAGs in our deletion strain compared to the parental strain (5). Similarly, Rashid et al. also noted accumulation of the LTA intermediate (glycerophospho-diglugosyl-diacylglcerol), GPDGDAG. As LTA is a component of the cell wall, such a compensatory mechanism could serve to reinforce the cellular structure; however, there was no observable difference in LTA content between strains via western analysis. This then begs the question of why are there so many glucosyl-derived lipids upon loss of L-PG without a subsequent increase in LTA? While viewed as anchors, these glucosyl-lipid species themselves will have impacts on membrane packing and fluidity, and detailed modeling studies may better address their role. It is also possible that the flux through lipid synthesis is occurring rapidly, and we may not be capturing the system completely. Additional microscopic analyses may reveal possible structural or morphological differences between the strains that may provide further clues as to *how* E. faecalis compensates for the loss of a lipid class.

Given such dramatic changes in the lipidome, it is not surprising that other physiological features are also impacted. Despite no obvious growth defects in rich media, there were significant differences in the transcriptome between a L-PG defective strain versus the parental strain. In particular, there was decreased expression of *de novo* fatty acid biosynthesis genes. Growth in defined media, lacking fatty acids, revealed a severe growth defect of Δ*mprF2* (and Δ*mprF1ΔmprF2*), the first such defect ever reported for these deletion strains in E. faecalis. Addition of either one of two saturated fatty acids present in rich media, palmitic acid (C_16:0_) or stearic acid (C_18:0_), improved growth of the Δ*mprF1ΔmprF2* strain in defined media. If fatty acid biosynthesis is reduced in the deletion strain(s), then addition of exogenous fatty acids could alleviate this via funneling those fatty acids directly into lipid synthesis (through a fatty acid kinase system, Fak) resulting in an increase placement of such tails on lipid headgroups (11, 12). Surprisingly, however, in the defined medium without fatty acid supplementation, the deletion strain had *higher* levels of palmitic acid in its membrane than the parental strain. Why would the strain depend upon exogenous saturated fatty acids if it is already elevated for such fatty acids? One possibility could be that these fatty acids are actually not being funneled appropriately to head groups and may be “free” within the membrane. Data from our group suggests that free fatty acids are present within the enterococcal membrane and perhaps they accumulate internally via the activity of a thioesterase or via environmental supplementation (4, 5, 13). Additionally, growth with exogenous fatty acids is known to repress expression of fatty acid biosynthesis genes in E. faecalis*:* these exogenous fatty acids are attached to ACP-B (acyl-carrier protein B) via a fatty acid kinase system (Fak). Acyl-ACP-B then interacts with the transcriptional repressor FabT, repressing fatty acid biosynthetic gene transcription (14). Reduced growth upon loss of L-PG is observed only in defined media that lacks fatty acids. This suggests an inherent metabolic problem with *de novo* fatty acid biosynthesis in the Δ*mprF2* strain that is relieved upon “shut-down” of this metabolic process. One possibility is that acyltransferases that are responsible for removing fatty acids from the fatty acid biosynthesis elongation cycle and transferring them to the phospholipid precursors are disrupted in activity. These acyltransferases associate with the membrane, and thus the change in lipid composition could greatly impact their abundance, activity or localization as has been seen with other membrane bound or associated proteins (some selected references [15–22]); such an idea is also reinforced via the secretion deficiencies observed here by Rashid et al.

It is clear that loss of L-PG has significant consequences for E. faecalis despite a lack of observable growth differences in rich media: however, is this just an anomaly for this particular lipid class in a single organism? We previously observed that detected CL species comprised a minor portion of total identified lipids for E. faecalis (1.1%). Yet, in a strain unable to produce CL (via deletion of its two synthase genes), there were significant alterations of the lipidome, including a large decrease in PG species with increase in DAGs; this strain was also highly sensitive to membrane and cell wall damaging agents (5). This further exemplifies that the loss of PG derivatives has significant impacts of PG recycling and/or synthesis and overall cellular physiology. Beyond E. faecalis, an elegant study in E. coli also noted that loss of independent phospholipid classes could have dramatic effects on cellular properties, yet the deletion strains were still viable within the lab (23). Clearly, loss of even “minor” lipid components results in complete metabolic rewiring to maintain membrane functionality.

The work by Rashid et al. is a clear reminder of the critical nature of the membrane and the significant metabolic rewiring cells will perform to maintain their integrity. Such a study can serve as a paradigm moving forward to aid in better understanding how the most fundamental feature of a cell, its membrane, shapes the entirety of cellular behavior.
